# Synthesis and biological evaluation of tricyclic matrinic derivatives as a class of novel anti-HCV agents

**DOI:** 10.1186/s13065-017-0327-8

**Published:** 2017-09-29

**Authors:** Sheng Tang, Zong-Gen Peng, Ying-Hong Li, Xin Zhang, Tian-Yun Fan, Jian-Dong Jiang, Yan-Xiang Wang, Dan-Qing Song

**Affiliations:** Institute of Medicinal Biotechnology, Chinese Academy of Medical Sciences and Peking Union Medical College, Beijing, 100050 China

**Keywords:** Matrinol, Hepatitis C virus, Structure–activity relationship, Druglike

## Abstract

**Background:**

12*N*-benzyl matrinic acid analogues had been identified to be a novel scaffold of anti-HCV agents with a specific mechanism, and the representative compound **1** demonstrated a moderate anti-HCV activity. The intensive structure–activity relationship of this kind of compounds is explored so as to obtain anti-HCV candidates with good druglike nature.

**Results:**

Taking compound **1** as the lead, 32 compounds (of which 27 were novel) with diverse structures on the 11-side chain, including methyl matrinate, matrinol, matrinic butane, (*Z*)-methyl Δ^βγ^-matrinic crotonate derivatives were synthesized and evaluated for their anti-HCV activities. Among all the compounds, matrinol **7a** demonstrated potential potency with a greatly improved SI value of 136. Pharmacokinetic studies of **7a** showed the potential for oral administration that would allow further in vivo safety studies. The free hydroxyl arm in **7a** made it possible to prepare pro-drugs for the potential in the treatment of HCV infection.

**Conclusions:**

27 novel 12*N*-substituted matrinol derivatives were prepared. The SAR study indicated that the introduction of electron-donating substitutions on the benzene ring was helpful for the anti-HCV activity, and the unsaturated 11-side chain might not be favorable for the activity. This study provided powerful information on further strategic optimization and development of this kind of compounds into a novel family of anti-HCV agents.

**Graphical abstract:**
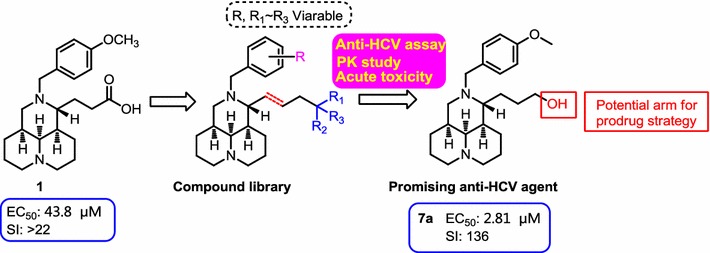
Matrinol derivatives as a class of novel anti-HCV agents

## Background

Currently, at least 130**–**150 million people worldwide have been infected with hepatitis C virus (HCV) [1]. Each year, 3**–**4 million people are newly infected and HCV-related liver complications kill estimated 700,000 people annually [1, 2]. In recent years, new direct acting antivirals (DAAs) specifically targeting HCV proteins have made a great breakthrough to HCV treatment, and NS3/4A HCV protease inhibitors telaprevir, boceprevir and simeprevir, NS5A inhibitors asunaprevir and ledipasvir, NS5B polymerase inhibitors sofosbuvir and dasabuvir have been approved by FDA for the HCV treatment successively since 2011 [3]. To deal with the springing up of drug resistance challenges [4–6], multiple of DAA combinations have been developed [7–9]. Therefore, it is still imperative to develop new anti-HCV agents with novel structure skeleton or mechanism of action as a new component to DAA combination.

**Fig. 1:**
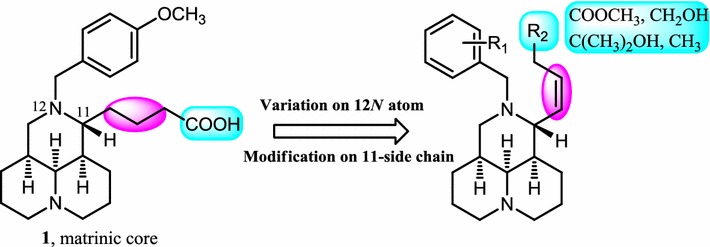
Modification sites based on compound **1**

In our earlier studies, 12*N*-benzyl matrinic acid analogues had been successfully identified to be a novel class of anti-HCV agents from matrine, a natural product extracted from traditional Chinese herb. The representative compound, 12*N*-4-methoxylbenzyl matrinic acid (**1**, Fig. 1) was identified to be active against HCV with a novel mechanism targeting on host protein Hsc70 and demonstrated a moderate anti-HCV activity with SI over 22 [10, 11]. The special tricyclic flexible scaffold and appealing druglike of compound **1** strongly provoked our interesting to continuously explore the structure–activity relationship (SAR) of this kind of compounds, in an effort to discover novel anti-HCV candidates which could be used in the combination with current DAA.

In the present study, as illustrated in Fig. 1, taking **1** as the lead, SAR studies were further conducted with the variations of the 11-side chain and diverse substituents on 12*N*-atom. Therefore, series of novel methyl matrinate, matrinol, matrinic butane, 1′, 1′-dialkyl matrinol, methyl (*Z*)-Δ^βγ^-matrinic crotonate and (*Z*)-Δ^βγ^-matrinic crotonl derivatives were designed, synthesized and evaluated for their in vitro anti-HCV activities as well as the in vivo pharmacokinetic (PK) and safety profile of the representative compounds.

## Results and discussion

### Chemistry

**Scheme 1:**
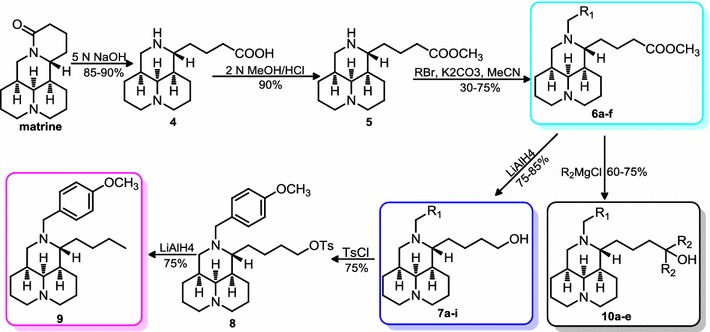
Synthetic procedures of methyl matrinate and matrinol derivatives. Reagents and conditions: (**a**) 5 N NaOH, reflux, 9 h, 6 N HCl, pH = 5–6; (**b**) 2 N MeOH/HCl, reflux, 2 h; (**c**) RBr, K_2_CO_3_, MeCN, r.t., overnight; (**d**) LiAlH_4_, THF, r.t., 30 min; (**e**) R_2_MgCl, THF, 0–25 °C, reflux, 2 h; (**f**) TsCl, CH_2_Cl_2_, TEA, 4-DMAP; (**g**) alkylmagnesium chloride, THF, reflux, 2 h

**Scheme 2:**
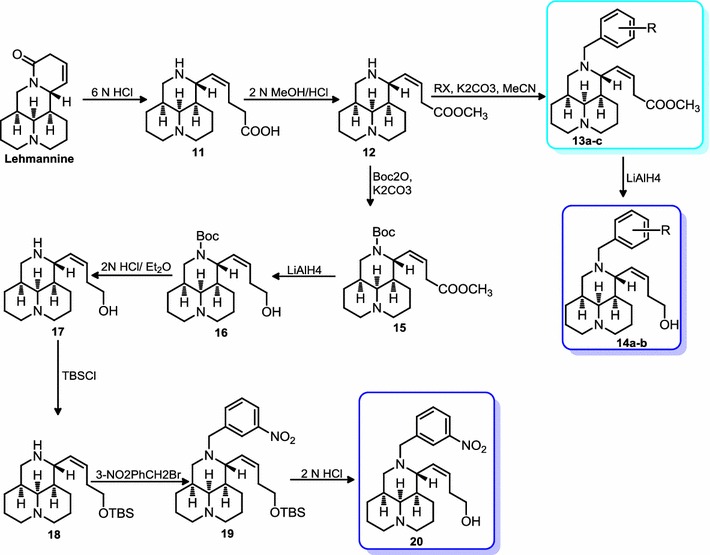
Synthetic procedures of methyl (*Z*)-Δ^βγ^-matrinic crotonate and (*Z*)-Δ^βγ^-matrinic crotonol derivatives. Reagents and conditions: (**a**) 6 N HCl, reflux, 9 h; (**b**) 2 N MeOH/HCl, reflux, 2 h; (**c**) RX, K_2_CO_3_, MeCN, r.t., overnight; (**d**) LiAlH_4_, THF, r.t., 30 min; (**e**) Boc_2_O, K_2_CO_3_, CH_2_Cl_2_, r.t., overnight; (**f**) 2 N HCl/Et_2_O, 30 min, (**g**) TBSCl, CH_2_Cl_2_, imidazole, r.t., overnight; (**h**) *3*-NO_2_PhCH_2_Br, TEA, CH_2_Cl_2_, r.t., 4 h; (**i**) 2 N HCl

As displayed in Schemes 1 and 2, all the target compounds were synthesized using commercially available matrine or lehmannine with purity over 98% as the starting material. As shown in Scheme 1, following the procedure of preparing compound **2** [12], the rest methyl matrinates **6a**–**f** were obtained from matrine through a three-step sequence including basic hydrolytic ring-opening, methyl esterification, 12*N*-substitution via substituted benzyl halides or benzaldehydes with good yields of 44–68% [13–17]. Similar to the preparation of **3a** [12], the rest matrinols **7a**–**i** were obtained by the LiAlH_4_ reduction of the corresponding methyl matrinate **6** as described in Scheme 1 with yields of 75–85%. The matrinic butane product **9** was achieved through hydroxyl sulfonylation, reductive-elimination of OTs by LiAlH_4_ from **7a** in a yield of 56% and the alkylation of **6a**–**b** and **6d**–**f** with Grignard reagents afforded the 1′,1′-dialkyl substituted matrinols **10a**–**e** in yields of 60–75% [12].

As depicted in Scheme 2, methyl (*Z*)-Δ^βγ^-matrinic crotonate derivatives (**13a**–**c**) were obtained from lehmannine following the similar sequence including acidic hydrolytic ring-opening, methyl esterification, 12*N*-substitution with overall yields of 30–35% [18]. The targeted (*Z*)-Δ^βγ^-matrinic crotonol derivatives (**14a**–**b**) were gained from a LiAlH_4_ reduction of **13a**–**b** in 70–80% yields. Another nitro substituted crotonol derivative **20** was obtained from compound **12** via a six-step procedure, including 12*N*-tert-butoxycarbonyl (Boc) protection, ester reduction by LiAlH_4_, de-protection of Boc, silicane protection, 12*N*-substitution and deprotection with an overall yield of 30% [14, 15].

### Anti-HCV activity and SAR analysis of matrinol derivatives

All the target compounds were evaluated for their anti-HCV activities (EC_50_) and cytotoxicities (CC_50_) in human Huh7.5 cells using specific real-time RT-PCR assay, as described earlier [11]. As an important indicator, the selectivity index (SI) was calculated as a ratio of CC_50_ to EC_50_. Anti-HCV ability of a given compound was estimated by combining its EC_50_ with SI values. Totally 32 compounds were gathered, and their structures and anti-HCV effects were shown in Table 1.

**Table 1:** SAR of all the targeted compounds for anti-HCV activity in Huh7.5 cells


Code	R_1_	R_2_	CC_50_ (μM)^a^	EC_50_ (μM)^b^	SI
**1**	C_6_H_4_OCH_3_-4	COOH	> 1000	43.8 ± 3.81	> 22.8
**2**	C_6_H_4_F-4	COOCH_3_	274 ± 8.35	2.09 ± 1.65	131
**3a**	C_6_H_4_F-4	CH_2_OH	285 ± 7.45	2.99 ± 1.51	95.3
**3b**	CH_2_CH_2_CH_3_	CH_2_OH	> 500	16.4 ± 10.9	> 30.5
**3c**	CH_2_(CH_2_)_5_CH_3_	CH_2_OH	33.3 ± 6.49	1.01 ± 0.55	32.9
**3d**	CH_2_(CH_2_)_6_CH_3_	CH_2_OH	17.8 ± 2.44	0.80 ± 0.26	22.3
**6a**	C_6_H_4_OCH_3_-4	COOCH_3_	414 ± 7.34	7.01 ± 1.51	59.1
**6b**	C_6_H_4_CH_3_-4	COOCH_3_	132.9 ± 7.53	1.73 ± 1.36	76.8
**6c**	C_6_H_4_CH = CH_2_-4	COOCH_3_	81.2 ± 12.9	1.61 ± 0.76	50.4
**6d**	C_6_H_3_F_2_-2,4	COOCH_3_	261 ± 28.7	1.69 ± 1.47	154
**6e**	C_6_H_4_NO_2_-4	COOCH_3_	235 ± 48.2	14.2 ± 3.13	16.5
**6f**	pyridyl-4	COOCH_3_	> 500	4.66 ± 0.35	> 107
**7a**	C_6_H_4_OCH_3_-4	CH_2_OH	383 ± 30.2	2.81 ± 0.82	136
**7b**	C_6_H_4_CH_3_-4	CH_2_OH	252 ± 3.04	< 2.06	> 122
**7c**	C_6_H_4_CH = CH_2_-4	CH_2_OH	143 ± 32.3	3.16 ± 0.51	45.3
**7d**	C_6_H_3_F_2_-2,4	CH_2_OH	266 ± 9.47	< 2.06	> 129
**7e**	Pyrid-4-yl	CH_2_OH	> 500	9.03 ± 5.58	> 55.4
**7f**	Pyrid-3-yl	CH_2_OH	> 500	4.39 ± 3.01	> 114
**7** **g**	pyrid-2-ylCl-5	CH_2_OH	> 500	10.9 ± 5.59	> 45.9
**7** **h**	CONHC_6_H_4_	CH_2_OH	> 500	46.7 ± 41.0	> 10.7
**7i**	CONHC_6_H_3_CF_3_-4	CH_2_OH	86.2 ± 4.33	4.19 ± 1.44	20.6
**9**			41.9 ± 1.10	1.16 ± 0.25	36.1
**10a**	C_6_H_4_OCH_3_-4	CH_3_	> 140 ± 16.6	11.7 ± 0.06	12.0
**10b**	C_6_H_4_CH_3_-4	CH_3_	55.7 ± 7.50	0.82 ± 0.26	67.9
**10c**	C_6_H_4_CH_3_-4	C_2_H_5_	12.3 ± 4.23	< 0.23	> 53.5
**10d**	C_6_H_3_F_2_-2,4	CH_3_	156 ± 37.9	6.55 ± 2.69	23.8
**10e**	Pyrid-4-yl	CH_3_	> 500	80.4 ± 24.3	6.22
**13a**	4-OCH_3_	COOCH_3_	326 ± 21.0	16.7 ± 4.71	19.5
**13b**	4-F	COOCH_3_	260 ± 84.8	18.1 ± 6.49	14.4
**13c**	3-NO_2_	COOCH_3_	402 ± 83.6	40.4 ± 3.64	10.0
**14a**	4-OCH_3_	CH_2_OH	214 ± 95.0	8.80 ± 2.15	24.3
**14b**	4-F	CH_2_OH	156 ± 65.8	13.6 ± 2.63	11.5
**20**	3-NO_2_	CH_2_OH	312 ± 31.1	18.4 ± 1.64	17.0
Tela			47.6 ± 0.61	0.02 ± 0.02	1950

*Tela* telaprevir

^a^Cytotoxic concentration required to inhibit Huh7.5 cell growth by 50%

^b^Concentration required to inhibit HCV growth by 50%

SAR investigation was initiated with the variation of carboxylic acid group, by which 7 methyl matrinates (**2**, **6a**–**f**) and 13 matrinols (**3a**–**d** and **7a**–**i**) were generated. As depicted in Table 1, except 4-nitrobenzyl derivative **6e**, all methyl 12*N*-benzyl/pyridylmethyl substituted matrinates exerted higher activities than the lead **1** by showing lower EC_50_ values and higher SI values of over 50. In particular, 12*N*-4-fluorobenzyl **2**, 4-methylbenzyl **6b**, 4-vinylbenzyl **6c** and 2,4-difluorobenzyl **6d** displayed potent anti-HCV activities with EC_50_ values ranging from 1.61 to 2.09 µM, which were over 20 times more potent than that of **1**. It appeared that the electron-donating substitutions on the benzene ring were more favorable than the electron-withdrawing groups in the methyl matrinate series.

Besides the substitutions mentioned above (**2**, **6a**–**d**, **6f**), 7 other substituents including long chain alkyl groups (**3b**–**d**), as well as pyridin-3-ylmethyl (**7f**), 5-chloropyridin-2-ylmethyl (**7g**), 2-oxo-2-(phenylamino)ethyl (**7h**), 2-oxo-2-((4- (trifluoromethyl) phenyl)amino)ethyl (**7i**) were also introduced on the 12*N* atom to generate the library of matrinols. As anticipated, most of the 12*N*-benzyl/pyridyl substituted matrinols (**3a** and **7a**–**g**) gave inspiring anti-HCV activities with EC_50_ values in the range of 2.06–10.9 μM, and SI values in the range of 45–136. In particular, compounds **7a**, **7b** and **7d** bearing electron-donating methoxy, methyl and 2,4-difluoro substitutions respectively gave excellent activities with EC_50_ values of less than 2.81 µM as well as SI values of over 122. However, alkyl (**3b**–**d**) or phenylamino carbonyl methyl compounds (**7h**–**i**) did not give favorable activities because of their either low activity or high cytotoxicity. It indicated again the favorability of electron-donating substitutions on the benzene ring to the anti-HCV activity.

Then, SAR investigation was focused on the influence of the structural type of the 11-side chain while the 12*N*-benzyl/pyridylmethyl substitution was retained. In the first round, matrinic butane (**9**), five 1′, 1′-dialkyl substituted matrinols (**10a**–**e**) were designed and synthesized. Among them, benzyl derived analogues (**9**, **10a**–**d**) exhibited promising anti-HCV activities with low micro molar EC_50_ values ranging from 0.23 to 11.70 μM, as well as limited toxicity with CC_50_ between 12.3 and 155.8 µM, while the 12*N*-pyrid-4-ylmethyl derivative **10e** showed a high EC_50_ value of 80.38 µM. The results indicated that 11-butane or 1′,1′-dialkyl butanol chain might not be helpful for the activity.

In the second round, to further examine the influence of saturation of 11-side chain on the activity, double bond was introduced to the β,γ position of the butyl acid chain, and the corresponding methyl Δ^βγ^-matrinic crotonates (**13a**–**c**) and crotonyl alcohols (**14a**–**b** and **20**) with 4-methoxyl, 4-fluoro, 4-nitrobenzyl substitution on the 12*N* atom were generated respectively. As described in Table 1, most compounds afforded very weak potencies with SI values between 10.0–24.3, inferring that the unsaturated side-chain might not be favorable for the HCV activity.

### PK study

**Fig. 2:**
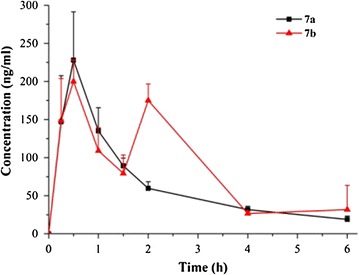
Mean plasma concentration-time profiles of the key compounds (25 mg kg^−1^, orally)

Based on above, methyl matrinates and matrinols exhibited the most potent anti-HCV activities, however, methyl matrinates might not possess favorable PK profiles in vivo owing to the exposed metabolically labile ester group. Therefore, two representative matrinols **7a** and **7b** were chosen to examine their PK parameters in SD rats at the single dosage of 25 mg kg^−1^ via oral route. As indicated in Table 2 and Fig. 2, both of them showed acceptable PK profiles with the areas under the curve (AUCs) of 1.58 and 2.36 μM**·**h and the half-times of 4.69 h and 3.39 h respectively, indicating reasonable stabilities in vivo. Meanwhile, the results demonstrated that the concentration of compounds **7a** and **7b** showed a significant difference at 2 h, owing to different dissolution rate at that time in vivo.

**Table 2:** PK parameters of the key compounds^a^

Code	Tmax (h)	Cmax (μM)	AUC 0–t (μM h)	AUC 0–∞ (μM h)	MRT (h)	t1/2 (h)
**7a**	0.42	0.62	1.55	1.58	3.55	4.69
**7b**	1.00	0.79	2.32	2.36	4.47	3.39

^a^PK parameters were calculated in rats after single oral dosing of 25 mg kg^−1^, (n = 3) by non-compartmental analysis using WinNonlin, version 5.3

### Acute toxicity study

The acute toxicity tests of **7a** and **7b** were performed in Kunming mice. Each compound was given orally in a single-dosing experiment at 250, 500, 750 or 1000 mg kg^−1^, respectively. The mice were closely monitored for 7 days. As indicated in Table 3, the LD_50_ values for **7a** and **7b** were 708 and 392 mg kg^−1^, respectively, therefore, **7a** seemed to be more promising as a parent drug from a safety prospective.

**Table 3:** Acute toxicity of the key compounds

Code	**7a**	**7b**
LD_50_ (mg kg^−1^)	708	392

## Experimental

### Instruments

Unless otherwise noted, all commercial reagents and solvents were obtained from the commercial provider and used without further purification. Melting points (mp) were obtained with CXM-300 melting point apparatus and are uncorrected. ^1^H NMR and ^13^C NMR spectra were recorded on a Bruker Avance 400 (400/101 MHz for ^1^H/^13^C) spectrometer or Bruker Avance III 500 (500/126 MHz for ^1^H/^13^C) spectrometer (Varian, San Francisco, USA) respectively, in DMSO-d_*6*_ with Me_4_Si as the internal standard. ESI high-resolution mass spectra (HRMS) were recorded on an AutospecUitima-TOF spectrometer (Micromass UK Ltd., Manchester, U.K.). Flash chromatography was performed on Combiflash Rf 200 (Teledyne, Nebraska, USA).

### General procedures for methyl 12*N*-substituted matrinate derivatives **6a**–**f**

Matrine (5.0 g, 20.0 mmol) was added to 5 N NaOH in water (30 mL), and the reaction mixture was refluxed for 9 h, cooled in an ice bath and then acidified with HCl (2 N) to pH 6–7. The solvent was removed in vacuo and the residue was dissolved with 2 N HCl in methanol and then heated at refluxing for 2 h. The solvent methanol was removed under reduced pressure to give crude **5** (5.5 g, yield 77%), which was applied directly in the next step without further purification.

To a stirred solution of **5** (10.0 mmol) and K_2_CO_3_ (35.0 mmol) in chloroethane (50 mL), the substituted benzyl halide (10 mmol) was added. The reaction mixture was stirred at room temperature for 5–8 h until TLC analysis showed completion of the reaction. Water (20 mL) was added to the mixture and the organic phase was separated and dried with anhydrous Na_2_SO_4_, concentrated, and the gained residue was purified by flash column chromatography on silica gel with CH_2_Cl_2_/CH_3_OH as the eluent to afford the title compounds.

#### Methyl 12*N*-(4-methoxybenzyl)matrinate dihydrochloride (**6a**)

The title compound was prepared from **5** and 4-methoxybenzyl bromide in the same manner as described above followed by an acidification with 2 N hydrochloride/ether (10 mL). Yield: 61%; white solid; mp 208–209 °C; ^1^H NMR (500 MHz) *δ* 11.42 (br, 1H), 11.06 (br, 1H), 7.53 (d, *J* = 8.7 Hz, 2H), 7.01 (d, *J* = 8.7 Hz, 2H), 4.93–4.89 (m, 1H), 4.22–4.18 (m, 1H), 4.00–3.88 (m, 2H), 3.79 (s, 3H), 3.61 (s, 3H), 3.58 (d, *J* = 10.4 Hz, 1H), 3.30–3.24 (m, 2H), 2.99–2.87 (m, 2H), 2.68–2.65 (m, 1H), 2.60–2.54 (m, 1H), 2.49–2.46 (m, 3H), 2.05–1.98 (m, 2H), 1.94–1.88 (m, 1H), 1.82–1.58 (m, 8H), 1.47 (d, *J* = 13.7 Hz, 1H); ^13^C NMR (126 MHz) *δ* 173.7, 160.3, 133.4 (2), 122.0, 114.6 (2), 60.7, 60.6, 57.2, 55.7, 54.7, 54.6, 51.8, 48.8, 36.3, 32.9, 30.4, 28.0, 24.5, 23.9, 21.8, 18.3 (2). HRMS: calcd for C_24_H_37_O_3_N_2_·2HCl [M−2HCl+H]^+^: 401.2799, found: 401.2790.

#### Methyl 12*N*-(4-methylbenzyl)matrinate (**6b**)

The title compound was prepared from **5** and 4-methylbenzyl bromide in the same manner as described in the general procedures. Yield: 67%; white solid; mp 89–91 °C; ^1^H NMR (500 MHz) *δ* 7.17 (d, *J* = 7.4 Hz, 2H), 7.10 (d, *J* = 7.4 Hz, 2H), 3.96 (d, *J* = 13.0 Hz, 1H), 3.55 (s, 3H), 3.01 (d, *J* = 12.9 Hz, 1H), 2.79 (s, 1H), 2.72 (d, *J* = 8.6 Hz, 1H), 2.65 (d, *J* = 9.1 Hz, 1H), 2.55 (d, *J* = 11.5 Hz, 1H), 2.30 (d, *J* = 6.3 Hz, 1H), 2.27 (s, 3H), 2.18 (d, *J* = 8.5 Hz, 1H), 1.96 (s, 1H), 1.85–1.24 (m, 17H); ^13^C NMR (126 MHz) *δ* 174.0, 137.6, 135.8, 129.2 (2), 128.9 (2), 64.3, 57.4, 57.1, 56.9, 55.4, 52.0, 51.6 (2), 37.6, 33.8, 28.4, 27.8, 27.4, 21.5, 21.2 (2), 19.0. HRMS: calcd for C_24_H_37_O_2_N_2_ [M+H]^+^: 385.2850, found: 385.2844.

#### Methyl 12*N*-(4-vinylbenzyl)matrinate (**6c**)

The title compound was prepared from **5** and 4-vinylbenzyl chloride in the same manner as **6b**. Yield: 70%; white solid; mp 75–77 °C. ^1^H NMR (500 MHz) *δ* 7.40 (d, *J* = 7.9 Hz, 2H), 7.27 (d, *J* = 7.9 Hz, 2H), 6.80–6.68 (m, 1H), 5.79 (d, *J* = 17.7 Hz, 1H), 5.21 (d, *J* = 11.1 Hz, 1H), 3.99 (d, *J* = 13.7 Hz, 1H), 3.54 (s, 3H), 3.10–3.01 (m, 1H), 2.88–2.78 (m, 1H), 2.78–2.67 (m, 1H), 2.70–2.60 (m, 1H), 2.63–2.56 (m, 1H), 2.29 (t, *J* = 6.7 Hz, 2H), 2.18 (d, *J* = 8.5 Hz, 1H), 1.96 (s, 1H), 1.85–1.52 (m, 11H), 1.36–1.24 (m, 5H); ^13^C NMR (126 MHz) *δ* 174.0, 140.7, 137.0, 135.9, 129.1 (2), 126.4 (2), 114.0, 64.3, 57.4, 57.1, 56.9, 55.4, 52.2, 51.6 (2), 37.6, 33.7, 28.3, 27.9, 27.4, 21.5, 21.2, 19.0. HRMS: calcd for C_25_H_37_O_2_N_2_ [M+H]^+^: 397.2850, found: 397.2838.

#### Methyl 12*N*-(2,4-difluorobenzyl)matrinate (**6d**)

The title compound was prepared from **5** and 2,4-difluorobenzyl bromide in the same manner as **6b**. Yield: 73%; white solid; mp 68–70 °C; ^1^H NMR (500 MHz) *δ* 7.48–7.43 (m, 1H), 7.18–7.14 (m, 1H), 7.08–7.04 (m, 1H), 3.95 (d, *J* = 13.8 Hz, 1H), 3.55 (s, 3H), 3.15 (d, *J* = 13.7 Hz, 1H), 2.85–2.83 (m, 1H), 2.72 (d, *J* = 10.7 Hz, 1H), 2.67–2.61 (m, 2H), 2.31–2.28 (m, 2H), 2.23–2.05 (m, 1H), 1.96 (s, 1H), 1.85–1.73 (m, 4H), 1.67–1.47 (m, 7H), 1.37–1.25 (m, 5H); ^13^C NMR (126 MHz) *δ* 173.9, 162.3, 160.3, 132.4, 123.4, 111.7, 103.9, 64.2, 57.3, 57.1 (2), 52.1, 51.6, 47.9, 37.6, 33.7, 33.6, 28.3, 27.9, 27.3, 21.5, 21.2, 19.1. HRMS: calcd for C_23_H_33_O_2_N_2_ F [M+H]^+^: 407.2505, found: 407.2488.

#### Methyl 12*N*-(4-nitrobenzyl)matrinate dihydrochloride (**6e**)

The title compound was prepared from **5** and 4-nitrobenzyl bromide in the same manner as **6a**. Yield: 75%; white solid; mp 215–217 °C; ^1^H NMR (500 MHz) *δ* 11.87 (br, 1H), 11.07 (br, 1H), 8.52–8.52 (m, 1H), 8.32–8.30 (m, 1H), 8.09 (d, *J* = 7.7 Hz, 1H), 7.77 (t, *J* = 8.0 Hz, 1H), 5.10 (d, *J* = 11.7 Hz, 1H), 4.27–4.23 (m, 1H), 4.22–4.16 (m, 1H), 4.00–3.93 (m, 1H), 3.61 (s, 3H), 3.60–3.56 (m, 1H), 3.35–3.10 (m, 2H), 3.00–2.87 (m, 2H), 2.82–2.77 (m, 1H), 2.61–2.57 (m, 1H), 2.53–2.37 (m, 2H), 2.12–1.51 (m, 13H); ^13^C NMR (126 MHz) *δ* 173.7, 148.3, 138.7, 132.2, 130.7, 126.9, 124.8, 60.8, 60.6, 56.6, 54.6, 51.8 (2), 49.2, 36.4, 32.9, 30.5, 28.0, 24.3, 23.9, 21.9, 18.3, 18.3. HRMS: calcd for C_23_H_34_O_4_N_3_·2HCl [M−2HCl+H]^+^: 416.2544, found: 416.2539.

#### Methyl 12*N*-(pyridin-4-ylmethyl)matrinate (**6f**)

The title compound was prepared from **5** and 4-(chloromethyl)pyridine in the same manner as **6b**. Yield: 45%; white solid; mp 84–86 °C; ^1^H NMR (500 MHz) *δ* 8.49–8.48 (m, 2H), 7.32 (d, *J* = 5.9 Hz, 2H), 4.02 (d, *J* = 14.7 Hz, 1H), 3.53 (s, 3H), 3.15 (d, *J* = 14.7 Hz, 1H), 2.90–2.87 (m, 1H), 2.74–2.64 (m, 3H), 2.29–2.26 (m, 2H), 2.25–2.08 (m, 1H), 1.98 (s, 1H), 1.85–1.76 (m, 4H), 1.65–1.25 (m, 12H); ^13^C NMR (126 MHz) *δ* 173.9, 150.3, 149.9 (2), 123.9 (2), 64.2, 57.3, 57.1, 56.7, 54.3, 52.6, 51.6, 37.6, 33.6, 33.5, 28.2, 27.8, 27.4, 21.5, 21.2, 19.0. HRMS: calcd for C_22_H_34_O_2_N_3_ [M+H]^+^: 372.2646, found: 372.2635.

### General procedures for 12*N*-substituted matrinol derivativess **7a**–**e**

A solution of LiAlH_4_ (12 mmol) in anhydrous THF (20 mL) was added to the solution of compound **6** (10 mmol) in anhydrous THF (3 mL) in an ice bath, the mixture solution was then stirred at room temperature for 30 min before the reaction was quenched with acetone. Saturated ammonium chloride (2 mL) was then added and the mixture was stirred for 30 min, and the precipitation was filtered off. The solvent was evaporated, and the residue was purified by flash column chromatography on silica gel with CH_2_Cl_2_/CH_3_OH as the eluent or followed by an acidification with 2 N hydrochloride/ether (10 mL) to afford target compounds.

#### 12*N*-(4-Methoxybenzyl)matrinol dihydrochloride (**7a**)

The title compound was prepared from **6a** as described above. Yield: 82%; white solid; mp 241–243 °C; ^1^H NMR (400 MHz) *δ* 11.04 (br, 1H), 10.99 (br, 1H), 7.52 (d, *J* = 8.7 Hz, 2H), 7.02 (d, *J* = 8.7 Hz, 2H), 4.82 (d, *J* = 11.2 Hz, 1H), 4.36 (s, 4H), 4.21–4.11 (m, 1H), 4.03–3.87 (m, 2H), 3.79 (s, 3H), 3.55 (d, *J* = 10.2 Hz, 1H), 3.27 (t, *J* = 13.0 Hz, 2H), 3.00–2.84 (m, 2H), 2.73–2.63 (m, 1H), 2.41 (d, *J* = 11.2 Hz, 1H), 1.92 (d, *J* = 9.3 Hz, 2H), 1.87–1.75 (m, 3H), 1.75–1.64 (m, 3H), 1.65–1.56 (m, 2H), 1.52 (s, 4H); ^13^C NMR (101 MHz) *δ* 159.9, 132.9 (2), 121.6 (2), 114.2, 60.4, 60.3, 60.1, 57.0, 55.2, 54.2, 54.2, 48.4, 36.1, 31.8, 30.0, 28.2, 24.1, 23.6, 22.7, 17.9, 17.8. HRMS: calcd for C_23_H_37_O_2_N_2_ ·2HCl [M−2HCl+H]^+^: 373.2850, found: 373.2848.

#### 12*N*-(4-Methylbenzyl)matrinol dihydrochloride (**7b**)

The title compound was prepared from **6b** as described above. Yield: 85%; white solid; mp 111–113 °C; ^1^H NMR (400 MHz) *δ* 11.20 (s, 1H), 11.06 (s, 1H), 7.48 (d, *J* = 8.0 Hz, 2H), 7.26 (d, *J* = 8.0 Hz, 2H), 4.85–4.80 (m 1H), 4.21–4.14 (m, 1H), 3.99–3.89 (m, 2H), 3.55 (d, *J* = 10.0 Hz, 1H), 3.26 (t, *J* = 13.6 Hz, 2H), 3.16 (s, 1H), 2.95 (m, 2H), 2.67–2.62 (m, 1H), 2.55 (m, 1H), 2.47–2.42 (m, 1H), 2.33 (s, 3H), 1.92–1.41 (m, 16H); ^13^C NMR (101 MHz) *δ* 138.8, 131.4 (2), 129.4 (2), 126.8, 60.6, 60.2, 60.1, 57.2, 54.2, 54.2, 48.6, 36.1, 31.8, 30.0, 28.2, 24.1, 23.6, 22.7, 20.8, 17.9, 17.8. HRMS: calcd for C_23_H_37_ON_2_·2HCl [M−2HCl+H]^+^: 357.2900, found: 357.2898.

#### 12*N*-(4-Vinylbenzyl)matrinol dihydrochloride (**7c**)

The title compound was prepared from **6c** as described above. Yield: 75%; white solid; mp 121–123 °C; ^1^H NMR (400 MHz) *δ* 11.02 (br, 2H), 7.92–7.50 (m, 4H), 6.78 (dd, *J* = 17.6, 10.8 Hz, 1H), 5.93 (d, *J* = 17.6 Hz, 1H), 5.34 (d, *J* = 11.2 Hz, 1H), 4.87 (d, *J* = 11.6 Hz, 1H), 3.54 (d, *J* = 10.0 Hz, 1H), 3.45 (m, 2H), 3.33–3.20 (m, 2H), 3.16 (s, 1H), 3.08–2.81 (m, 3H), 2.78–2.57 (m, 2H), 2.45–2.40 (m, 1H), 2.11–1.33 (m, 16H); ^13^C NMR (101 MHz) *δ* 138.0, 136.0, 131.7 (2), 129.3, 126.4 (2), 115.6, 60.6, 60.2, 60.1, 57.2, 54.2, 48.7, 36.1, 31.8, 30.0, 28.2, 24.0, 23.6, 22.7, 18.6, 17.9, 17.8. HRMS: calcd for C_24_H_37_ON_2_·2HCl [M−2HCl+H]^+^: 369.2900, found: 369.2900.

#### 12*N*-(2,4-Difluorobenzyl)matrinol dihydrochloride (**7d**)

The title compound was prepared from **6d** as described above. Yield: 80%; white solid, mp 124–126 °C; ^1^H NMR (400 MHz) *δ* 11.13 (br, 1H), 10.87 (br, 1H), 7.92–7.80 (m, 1H),7.45–7.38 (m, 1H), 7.28–7.18 (m, 1H), 4.77 (d, *J* = 13.0 Hz, 1H), 4.28–4.08 (m, 2H), 4.08–3.92 (m, 2H), 3.54 (d, *J* = 10.2 Hz, 2H), 3.28 (t, *J* = 12.0 Hz, 2H), 2.99–2.87 (m, 4H), 2.42–2.38 (m, 1H), 2.02–1.87 (m, 3H), 1.87–1.83 (m, 2H), 1.79–1.68 (m, 3H), 1.68–1.58 (m, 3H), 1.52 (s, 4H); ^13^C NMR (101 MHz) *δ* 135.5, 132.6, 113.4, 112.1, 111.9, 104.4, 60.4, 60.1, 60.1, 54.2, 54.2, 49.8, 48.7, 36.0, 31.8, 30.0, 28.1, 23.9, 23.7, 22.4, 17.9, 17.8. HRMS: calcd for C_22_H_33_ON_2_F_2_·2HCl [M−2HCl+H]^+^: 379.2556, found: 379.2551.

#### 12*N*-(Pyridin-4-ylmethyl)matrinol dihydrochloride (**7e**)

The title compound was prepared from **6f** as described above. Yield: 77%; white solid; mp 205–206 °C; ^1^H NMR (400 MHz) *δ* 12.40 (br, 1H), 11.08 (br, 1H), 9.00 (d, *J* = 5.5 Hz, 2H), 8.34 (d, *J* = 5.5 Hz, 2H), 5.17 (s, 1H), 4.42–4.16 (m, 2H), 3.99–3.95 (m, 1H), 3.62 (d, *J* = 9.7 Hz, 1H), 3.43 (t, *J* = 5.6 Hz, 2H), 3.30–3.17 (m, 3H), 2.95–2.89 (m, 3H), 2.71 (d, *J* = 9.8 Hz, 1H), 2.07 (s, 1H), 1.95–1.39 (m, 14H); ^13^C NMR (101 MHz) *δ* 148.3 (2), 143.5, 129.4 (2), 61.4, 60.6, 56.1, 54.6, 49.9,49.0, 39.6 (2), 36.5, 32.3, 30.5, 28.8, 24.2, 24.1, 23.3, 18.3. HRMS: calcd for C_21_H_34_ON_3_·2HCl [M−2HCl+H]^+^: 344.2696, found: 344.2694.

### General procedures for 12*N*-substituted matrinol derivatives **7f**–**i**

To a stirred solution of **5** (5.0 mmol) and K_2_CO_3_ (17.0 mmol) in dichloroethane (50 mL), substituted pyridylmethyl halide or phenylcarbamic chloride (5 mmol) was added. The reaction mixture was stirred at room temperature for 8 h until TLC analysis showed completion of the reaction. Water (20 mL) was added to the mixture and the organic phase was separated and dried with anhydrous Na_2_SO_4_, concentrated. To a solution of the gained residue in anhydrous THF (3 mL) in an ice bath, a solution of LiAlH_4_ (6 mmol) in anhydrous THF (10 mL) was added, the mixture solution was stirred at room temperature for 30 min before the reaction was quenched with acetone. The saturated ammonium chloride (2 mL) was then added and the mixture was stirred for 30 min, and the precipitation was filtered off. Then the solvent was evaporated, and the residue was purified by flash column chromatography on silica gel with CH_2_Cl_2_/CH_3_OH as the eluent to afford the target compounds.

#### 12*N*-(Pyridin-3-ylmethyl)matrinol (**7f**)

The title compound was prepared from **5** and *3*-chloromethylpyridine as described above. Yield: 43%; yellow oil; ^1^H NMR *δ* (500 MHz) 9.13 (s, 1H), 8.96 (d, *J* = 5.5 Hz, 1H), 8.78 (d, *J* = 5.5 Hz, 1H), 8.07 (t, *J* = 5.5 Hz, 1H), 5.04–4.97(m, 1H), 4.37–4.17 (m, 2H), 3.95–3.92 (m, 1H), 3.64–3.62 (m, 1H), 3.45 (t, *J* = 5.9 Hz, 2H), 3.29–3.18 (m, 3H), 3.07–2.86 (m, 4H), 2.68–2.66 (m, 1H), 2.09–2.08 (m, 1H), 1.93–1.91 (m, 2H), 1.86–1.47 (m, 11H); ^13^C NMR (126 MHz) *δ* 148.1, 145.8, 143.8, 129.5, 127.1, 61.2, 60.8, 60.6, 54.6, 53.8, 49.8, 49.6, 36.6, 32.3 (2), 30.6, 28.5, 24.3, 24.1, 22.9, 18.3. HRMS: calcd for C_21_H_34_ON_3_ [M+H]^+^: 344.2696, found: 344.2694.

#### 12*N*-(5-Chloropyridin-2-ylmethyl)matrinol dihydrochloride (**7g**)

The title compound was prepared from **5** and *5*-chloro-*2*-(chloromethyl)pyridine in the same manner as **7f** followed by an acidification with 2 N hydrochloride/ether (3 mL). Yield: 48%; light yellow solid; mp: 91–92 °C; ^1^H NMR (500 MHz) *δ* 11.98 (br, 1H), 11.06 (br, 1H), 8.61 (d, *J* = 2.4 Hz, 1H), 8.19 (dd, *J* = 8.2, 2.4 Hz, 1H), 7.64 (d, *J* = 8.2 Hz, 1H), 5.01–4.95 (m, 1H), 4.33–4.22 (m, 2H), 3.99–3.95 (m, 1H), 3.64–3.62 (m, 1H), 3.43–3.41 (m, 2H), 3.30–3.17 (m, 3H), 2.95–2.89 (m, 3H), 2.71 (d, *J* = 9.8 Hz, 1H), 2.07 (s, 1H), 1.97–1.35 (m, 14H); ^13^C NMR (126 MHz) *δ* 152.9, 151.7, 143.3, 125.8, 124.8, 60.7, 60.3, 54.7, 53.9, 51.8, 49.2, 39.5, 36.5, 33.2, 32.9, 30.5, 28.0, 24.3, 23.9, 21.5, 18.4. HRMS: calcd for C_21_H_33_ON_3_Cl·2HCl [M−2HCl+H]^+^: 378.2307, found: 378.2304.

#### 12*N*-(2-Oxo-2-(phenylamino)ethyl)matrinol (**7h**)

The title compound was prepared from **5** and phenylcarbamic chloride in the same manner as **7f**. Yield: 46%; white solid; mp: 136–137 °C; ^1^H NMR (400 MHz) *δ* 9.64 (br, 1H), 7.64–7.57 (m, 2H), 7.31 (t, *J* = 7.9 Hz, 2H), 7.06 (t, *J* = 7.4 Hz, 1H), 4.41–4.25 (m, 1H), 3.41–3.37 (m, 2H), 3.03 (s, 2H), 2.75–2.72 (m, 2H), 2.44–2.31 (m, 1H), 2.00–1.93 (m, 2H), 1.85–1.76 (m, 3H), 1.70–1.48 (m, 4H), 1.47–1.20 (m, 12H); ^13^C NMR (101 MHz) *δ* 170.1, 138.9, 131.1 (2), 123.9, 119.8 (2), 64.2, 61.2, 61.1, 57.3, 56.4, 55.7, 53.9, 37.9, 33.2, 29.7 (2), 28.7, 28.1, 27.4, 21.5, 20.8. HRMS: calcd for C_23_H_36_O_2_N_3_ [M+H]^+^: 386.2802, found: 386.2800.

#### 12*N*-(2-Oxo-2-((4-(trifluoromethyl)phenyl)amino)ethyl)matrinol dihydrochloride (**7i**)

The title compound was prepared from **5** and *4*-(trifluoromethyl)phenylcarbamic chloride in the same manner as **7** **g**. Yield: 62%; white solid; mp: 185–187 °C; ^1^H NMR (400 MHz) *δ* 11.20 (br, 1H), 10.48 (br, 1H), 10.08 (br, 1H), 8.44 (d, *J* = 1.6 Hz, 1H), 7.56–7.46 (m, 1H), 7.30 (d, *J* = 8.6 Hz, 1H), 4.65–4.55 (m, 1H), 4.32–4.16 (m, 2H), 4.09 (d, *J* = 9.4 Hz, 1H), 3.46–3.30 (m, 2H), 3.26 (t, *J* = 9.5 Hz, 2H), 3.03–2.87 (m, 2H), 2.60–2.56 (m, 1H), 2.46–2.42 (m, 1H), 1.95–1.60 (m, 12H), 1.50–1.30 (m, 6H); ^13^C NMR (101 MHz) *δ* 164.5, 152.8, 127.2 (2), 126.2, 118.9 (2), 61.2, 60.7, 60.4, 56.8, 54.7, 54.6, 52.3, 36.6, 32.3, 30.7, 29.7, 29.2, 24.3, 24.1, 23.7, 18.4, 18.3. HRMS: calcd for C_24_H_35_O_2_N_3_F_3_·2HCl [M−2HCl+H]^+^: 454.2676, found: 454.2679.

### Synthesis of 12*N*-4-methoxybenzyl matrinic butane **9**

To a solution of **7a** (5 mmol) in anhydrous CH_2_Cl_2_ (20 mL), TsCl (5 mmol), TEA (10 mmol) and dimethylamino pyridine (0.5 mmol) were added and stirred at room temperature until the TLC showed completion of the reaction. The solution was washed successively by water (10 mL), saturated ammonium chloride solution (10 mL) and brine (10 mL), dried over anhydrous sodium sulfate, and concentrated to obtain crude **8**. To a solution of the crude **8** in anhydrous THF, a solution of LiAlH_4_ (6 mmol) in anhydrous THF was added in an ice bath, then the mixture was stirred at room temperature for 30 min, the reaction was then quenched with acetone, 2 ml saturated ammonium chloride was added and stirred for 30 min, and the precipitation was filtrated. The gained residue was purified by flash column chromatography on silica gel with CH_2_Cl_2_/CH_3_OH as the eluent to afford the title compound **9** as a yellow solid. Yield: 56%; mp: 73–74 °C; ^1^H NMR (400 MHz) *δ* 7.22 (d, *J* = 8.8 Hz, 2H), 6.88 (d, *J* = 8.8 Hz, 2H), 3.93–3.88 (m, 1H), 3.74 (s, 3H), 3.43–3.21 (m, 1H), 3.00 (d, *J* = 10.3 Hz, 1H), 2.88–2.61 (m, 3H), 2.53 (d, *J* = 11.7 Hz, 1H), 2.22-2.15 (m, 1H), 1.96 (s, 1H), 1.90–1.73 (m, 3H), 1.64 (s, 2H), 1.50-1.37 (m, 12H), 0.87 (s, 3H); ^13^C NMR (101 MHz) *δ* 158.4, 129.9, 128.4, 114.1 (2), 113.9, 64.3, 63.0, 57.1, 55.5, 55.4, 55.1, 52.0, 37.8, 37.5, 33.7, 28.4, 27.5, 25.9, 23.0, 21.6, 21.2, 14.5. HRMS: calcd for C_23_H_37_ON_2_ [M+H]^+^: 357.2900, found: 357.2899.

### General procedures for 1′,1′-dialkyl-12*N*-substituted matrinol derivatives **10a**–**e**

To a solution of compound **6** (5 mmol) in anhydrous THF (10 mL), a solution of 2 *N* alkylmagnesium chloride in THF (25 mmol) was added in an ice bath, and the mixture solution was heated at refluxing for 2 h. After reaction completed, the reaction was quenched with a solution of saturated aqueous ammonium chloride (2 mL). The residue was purified by flash column chromatography on silica gel with CH_2_Cl_2_/CH_3_OH as the eluent followed by the acidification with 2 N hydrochloride/ether (3 mL) to afford the title compounds.

#### 1′,1′-Dimethyl-12*N*-(4-methoxybenzyl)matrinol dihydrochloride (**10a**)

The title compound was prepared from **6a** and methylmagnesium chloride using the same method as described above. Yield: 67%; white solid; mp: 125–127 °C; ^1^H NMR (400 MHz) *δ* 11.35 (br, 1H), 11.05 (br, 1H), 7.53 (d, *J* = 8.8 Hz, 2H), 6.99 (d, *J* = 8.8 Hz, 2H), 4.78 (d, *J* = 11.2 Hz, 1H), 4.22–4.12 (m, 1H), 3.94–3.89 (m, 2H), 3.77 (s, 3H), 3.59 (d, *J* = 10.0 Hz, 1H), 3.25 (t, *J* = 13.6 Hz, 2H), 3.00–2.87 (m, 2H), 2.68–2.57 (m, 2H), 2.46 (d, *J* = 12.4 Hz, 1H), 2.02–1.51 (m, 12H), 1.46–1.39 (m, 4H), 1.11-1.07 (m, 5H); ^13^C NMR (126 MHz) *δ* 159.8, 132.9 (2), 121.6, 114.1 (2), 72.4, 68.7, 57.0, 55.2, 54.2, 54.1, 44.8, 42.9, 35.7, 32.3, 32.0, 29.9, 29.6, 29.2, 27.6, 25.5, 24.1, 23.6, 21.3. HRMS: calcd for C_25_H_41_O_2_N_2_·2HCl [M−2HCl+H]^+^: 401.3163, found: 401.3163.

#### 1′,1′-Dimethyl-12*N*-(4-methylbenzyl)matrinol dihydrochloride (**10b**)

The title compound was prepared from **6b** and methylmagnesium chloride using the same method as described above. Yield 63%; white solid; mp: 120–122 °C; ^1^H NMR (400 MHz) *δ* 11.12 (br, 1H), 10.98 (br, 1H), 7.48 (dd, *J* = 8.0, 5.6 Hz, 2H), 7.27 (d, *J* = 7.6 Hz, 2H), 4.91–4.74 (m, 1H), 4.33–4.14 (m, 2H), 4.04 (s, 5H), 3.62–3.54 (m, 1H), 3.29–3.24 (m, 2H), 2.99–2.88 (m, 2H), 2.73–2.68 (m, 1H), 2.34 (s, 3H), 2.04–1.56 (m, 15H), 1.49–1.42 (m, 2H), 1.10 (d, *J* = 3.2 Hz, 2H); ^13^C NMR (126 MHz) *δ* 132.1, 123.1 (2), 121.5 (2), 117.9, 62.3, 61.7, 53.3, 53.0, 50.1, 46.9, 41.5, 36.8, 34.4, 28.4, 23.4, 23.3, 22.9 (2), 19.9 (2), 15.9, 15.6, 11.8. HRMS: calcd for C_25_H_41_ON_2_·2HCl [M−2HCl+H]^+^: 385.3213, found: 385.3214.

#### 1′,1′-Diethyl-12*N*-(4-methylbenzyl)matrinol dihydrochloride (**10c**)

The title compound was prepared from **6b** and ethylmagnesium chloride using the same method as described above. Yield 72%; yellow oil; ^1^H NMR (400 MHz) *δ* 10.91 (br, 1H), 10.54 (br, 1H), 7.45 (d, *J* = 8.0 Hz, 2H), 7.29 (d, *J* = 7.6 Hz, 2H), 4.85–4.80 (m, 1H), 4.56–4.14 (m, 3H), 4.06–3.82 (m, 3H), 3.61–3.53 (m, 3H), 3.35–3.24 (m, 2H), 3.12–2.85 (m, 3H), 2.78 (d, *J* = 6.8 Hz, 1H), 2.34 (s, 3H), 2.09–1.99 (m, 3H), 1.95–1.69 (m, 9H), 1.69–1.53 (m, 3H), 1.53–1.33 (m, 2H), 0.96 –0.92 (m, 4H); ^13^C NMR (126 MHz) *δ* 138.9, 131.3 (2), 129.3 (2), 126.8, 80.6, 60.3, 60.2, 57.3, 54.2 (2), 48.9, 35.6, 32.9, 32.8, 30.0, 24.0, 23.6, 20.8 (2), 19.9, 17.8, 13.2, 12.6, 8.6 (2). HRMS: calcd for C_27_H_45_ON_2_·2HCl [M−2HCl+H]^+^: 413.3526, found: 413.3524.

#### 1′,1′-Dimethyl-12*N*-(2,4-difluorobenzyl)matrinol dihydrochloride (**10d**)

The title compound was prepared from **6d** and methylmagnesium chloride using the same method as described above. Yield: 75%; white solid; mp: 237–238 °C; ^1^H NMR (400 MHz) *δ* 11.02 (s, 1H), 10.40 (s, 1H), 7.93–7.81 (m, 1H),7.47–7.40 (m, 1H), 7.29–7.19 (m, 1H), 4.78 (d, *J* = 13.2 Hz, 1H), 4.30–4.20 (m, 1H), 4.20–4.05 (m, 1H), 4.10–3.85 (m, 1H), 3.53 (d, *J* = 8.4 Hz, 3H), 3.29 (t, *J* = 12.0 Hz, 2H), 3.06–2.82 (m, 4H), 2.00–1.51 (m, 11H), 1.47–1.44 (m, 3H), 1.11 (d, *J* = 3.6 Hz, 6H); ^13^C NMR (126 MHz) *δ* 164.2, 162.2, 135.6, 113.5, 112.0, 104.3, 68.6, 60.5, 60.1, 54.2 (2), 49.8, 48.8, 42.9, 35.9, 29.9, 29.5, 29.2, 28.7, 23.9, 23.7, 20.4, 17.8 (2). HRMS: calcd for C_24_H_37_ON_2_F_2_·2HCl [M−2HCl+H]^+^: 407.2868, found: 407.2863.

#### 1′,1′-Dimethyl-12*N*-(4-pyridylmethyl)matrinol (**10e**)

The title compound was prepared from **6f** and methylmagnesium chloride using the same method as described above without acidification. Yield: 68%; yellow solid; mp: 243–245 °C; ^1^H NMR (400 MHz) *δ* 8.47 (d, *J* = 5.6 Hz, 2H), 7.33 (d, *J* = 5.6 Hz, 2H), 4.02 (s, 1H), 3.94 (d, *J* = 15.2 Hz, 1H), 3.32 (s, 1H), 3.19 (d, *J* = 14.4 Hz, 1H), 2.89 (d, *J* = 8.4 Hz, 1H), 2.72 (t, *J* = 11.2 Hz, 3H), 2.17 (d, *J* = 8.8 Hz, 1H), 2.00 (s, 1H), 1.81 (d, *J* = 12.0 Hz, 3H), 1.64–1.54 (m, 4H), 1.42–1.24 (m, 10H), 1.01 (d, *J* = 2.8 Hz, 6H); ^13^C NMR (126 MHz) *δ* 149.7, 149.4 (2), 123.3 (2), 68.7, 63.8, 56.5, 56.4, 53.7, 51.9, 43.9, 37.0, 34.7, 32.7, 29.4, 29.1, 29.0, 27.4, 26.7, 20.8, 20.5, 18.1. HRMS: calcd for C_23_H_38_ON_3_ [M+H]^+^: 372.3009, found: 372.3008.

### General procedures for methyl (*Z*)-12*N*-substituted Δ^βγ^-matrinic crotonate derivatives **13a**–**c**

Lehmannine (3.0 g, 12.2 mmol) was added to a solution of 5 N HCl (30 mL). The reaction mixture was heated at reflux for 9 h. The solvent was then removed *in vacuo*, and the residue was recrystallized by methanol and ethyl acetate to afford the intermediate **11** (2.5 g, 60%) as white solid. mp: 191–193 °C. ^1^H NMR (400 MHz) *δ* 12.39 (s, 1H), 11.21 (d, *J* = 8.0 Hz, 1H), 10.27 (d, *J* = 9.3 Hz, 1H), 9.30 (d, *J* = 9.0 Hz, 1H), 6.01 (dt, *J* = 10.8, 7.3 Hz, 1H), 5.49 (t, *J* = 10.4 Hz, 1H), 5.04–4.92 (m, 1H), 3.99–3.764 (m, 1H), 3.65 (d, *J* = 10.1 Hz, 1H), 3.44–3.33 (m, 2H), 3.25–3.20 (m, 2H), 3.20–3.02 (m, 1H), 2.97–2.89 (m, 2H), 2.55–2.51 (m, 1H), 2.40–2.23 (m, 1H), 1.89–1.56 (m, 8H); ^13^C NMR (101 MHz) *δ* 172.4, 132.4, 125.7, 60.4, 54.8, 54.7, 49.8, 41.5, 35.5, 33.8, 30.8, 24.6, 23.6, 18.5(2); HRMS: calcd for C_15_H_25_N_2_O_2_·2HCl [M−2HCl+H]^+^: 265.1911, found: 265.1909.

Compound **11** (1.0 g, 3.0 mmol) was dissolved in 2 N MeOH/HCl (30 mL), and the reaction mixture was refluxed for 2 h. Compound **12** was obtained by evaporation and used in the next reaction without further purification. Anhydrous K_2_CO_3_ (3.5 equiv) and substituted benzyl bromide (1.5 equiv) were added to a solution of compound **12** in acetonitrile (30 mL), and the reaction solution was then stirred at room temperature until TLC analysis showed completion of the reaction. The reaction mixture was filtered, and the filtrate was washed by water and brine, dried with anhydrous Na_2_SO_4_, filtrated, and concentrated to afford crude compound **13**. The title compounds were obtained by purifying with flash column chromatography on silica gel with dichloromethane and methanol as the eluent.

#### (*Z*)-Methyl 12*N*-(4-methoxybenzyl)-Δ^βγ^-matrinic crotonate (**13a**)

The title compound was prepared from **12** and 4-methoxybenzyl bromide using the same method as described above. Yield: 62%; white solid; mp: 98–100 °C. ^1^H NMR (400 MHz) *δ* 7.14 (d, *J* = 8.4 Hz, 2H), 6.84 (d, *J* = 8.4 Hz, 2H), 5.81–5.75 (m, 1H), 5.33 (t, *J* = 10.4 Hz, 1H), 3.92 (d, *J* = 13.2 Hz, 1H), 3.73 (s, 3H), 3.61 (s, 3H), 3.34–3.15 (m, 3H), 2.89–2.86 (m, 1H), 2.72–2.75(m, 2H), 2.51–2.44 (m, 1H), 2.21–2.18 (m, 1H), 1.98 (s, 1H),1.84–1.75 (m, 2H), 1.65–1.24 (m, 10H); ^13^C NMR (126 MHz) *δ* 171.3, 158.0, 136.0, 131.5, 129.7 (2), 124.8, 113.4 (2), 62.5, 58.2, 56.8, 56.7, 54.9 (2), 51.6 (2), 50.8, 34.7, 33.3, 28.1, 26.8, 21.4, 21.2. HRMS: calcd for C_24_H_35_N_2_O_3_ [M+H]^+^: 399.2642, found: 399.2642.

#### (*Z*)-Methyl 12*N*-(4-fluorobenzyl)-Δ^βγ^-matrinic crotonate dihydrochloride (**13b**)

The title compound was prepared from **12** and 4-fluorobenzyl bromide using the same method as described above. Yield: 68%; white solid; mp: 151–153 °C. MS–ESI m/s: 387; ^1^H NMR (400 MHz) *δ* 11.93 (d, *J* = 8.0 Hz, 1H), 11.08 (d, *J* = 7.6 Hz, 1H), 7.64–7.61 (m, 2H), 7.32–7.27 (m, 2H), 6.27–6.20 (m, 1H), 5.88–5.78 (m, 1H), 5.28 (t, *J* = 11.2 Hz, 1H), 4.63 (d, *J* = 12.0 Hz, 1H), 4.00–3.85 (m, 2H), 3.68–3.17 (m, 9H), 3.00–2.79 (m, 3H), 2.63 (s, 1H), 1.83–1.56 (m, 8H); ^13^C NMR (126 MHz) *δ* 170.7, 161.7, 133.7, 133.6, 133.2, 126.0, 124.9, 115.9, 115.8, 59.6, 58.6, 56.7, 54.2, 54.1, 51.9, 47.3, 35.1, 33.3, 30.2, 24.0, 23.9, 18.0, 17.9. HRMS: calcd for C_23_H_32_FN_2_O_2_·2HCl [M−2HCl+H]^+^: 387.2442, found: 387.2446.

#### (*Z*)-Methyl 12*N*-(3-nitrobenzyl)-Δ^βγ^-matrinic crotonate dihydrochloride (**13c**)

The title compound was prepared from **12** and 3-nitrobenzyl bromide using the same method as described above. Yield: 70%; white solid; mp: 185–187 °C; ^1^H NMR (400 MHz) *δ* 12.33 (s, 1H), 11.07 (s, 1H), 8.41 (s, 1H), 8.26 (d, *J* = 8.4 Hz, 1H), 8.02 (d, *J* = 7.5 Hz, 1H), 7.71 (t, *J* = 8.0 Hz, 1H), 6.22 (dt, *J* = 15.1, 7.5 Hz, 1H), 5.84 (t, *J* = 10.6 Hz, 1H), 5.39–5.21 (m, 1H), 4.70 (d, *J* = 12.9 Hz, 1H), 4.15–3.78 (m, 3H), 3.64 (s, 3H), 3.47–3.41 (m, 1H), 3.26 (d, *J* = 11.7 Hz, 2H), 2.99–2.83 (m, 3H), 2.61 (s, 1H), 2.56–2.48 (m, 1H), 1.80–1.76 (m, 2H), 1.70–1.49 (m, 7H); ^13^C NMR (126 MHz) *δ* 170.7, 147.8, 138.1, 133.4, 131.6, 130.4, 126.3, 124.9, 124.4, 59.6, 58.7, 56.5, 54.3, 54.1, 51.9, 47.7, 35.2, 33.3, 30.3, 23.9, 23.9, 18.0, 17.9. HRMS: calcd for C_23_H_32_N_3_O_4_·2HCl [M−2HCl+H]^+^: 414.2387, found: 414.2391.

### General procedures for (*Z*)-12*N*-substituted Δ^βγ^-matrinic crotonol derivatives **14a**–**b**

A solution of the LiAlH_4_ in THF (2.4 N, 1.2 equiv) was added to the solution of compound **13** in anhydrous THF in ice bath, then the mixture solution was stirred at room temperature for 30 min, the reaction was then quenched with acetone, 2 mL saturated ammonium chloride solution was added and stirred for 30 min, and the precipitation was filtrated off. The filtrate was concentrated, and the residue was purified by flash column chromatography on silica gel with dichloromethane and methanol as the eluent followed by the acidification by 2 N hydrochloride/ether to afford compounds.

#### (*Z*)-12*N*-(4-Methoxybenzyl)-Δ^βγ^-matrinic crotonol dihydrochloride (**14a**)

The title compound was prepared from **13a** using the same method as described above. Yield: 86%; white solid; mp: 175–177 °C; ^1^H NMR (400 MHz) *δ* 11.46 (br, 1H), 11.15 (br, 1H), 7.48–7.45 (d, *J* = 8.8 Hz, 2H), 7.01–6.99 (d, *J* = 8.8 Hz, 2H), 6.17–6.11 (m, 1H), 5.68 (t, *J* = 10.8 Hz, 1H), 5.16 (dd, *J* = 10.4 Hz, 1H), 4.66 (d, *J* = 11.6 Hz, 1H), 4.02–3.93 (m, 1H), 3.89–3.78 (m, 1H), 3.74 (s, 3H), 3.69–3.43 (m, 5H), 3.39–3.28 (m, 3H), 2.99–2.88 (m, 2H), 2.78–2.75 (m, 1H), 2.55–2.49 (m, 1H), 2.37–2.31 (m, 1H), 1.89–1.53 (m, 8H); ^13^C NMR (126 MHz) *δ* 159.9, 139.2, 132.8 (2), 123.5, 121.6, 114.2 (2), 59.8, 59.7, 58.6, 57.1, 55.2 (2), 54.2, 47.1, 35.3, 31.8, 30.2, 24.2, 24.0, 18.0, 17.9. HRMS: calcd for C_23_H_35_N_2_O_2_·2HCl [M−2HCl+H]^+^: 371.2693, found: 371.2698.

#### (*Z*)-12*N*-4-(Fluorobenzyl)-Δ^βγ^-matrinic crotonol dihydrochloride (**14b**)

The title compound was prepared from **13b** using the same method as described above. Yield: 87%; white solid; mp: 194–196 °C; ^1^H NMR (400 MHz) *δ* 11.77 (br, 1H), 11.15 (br, 1H), 7.64 (dd, *J* = 5.6 Hz, 2H), 7.28 (t, *J* = 8.8 Hz, 2H), 6.18–6.11 (m, 1H), 5.71 (t, *J* = 10.8 Hz, 1H), 5.18 (dd, *J* = 10.8 Hz, 1H), 4.70 (d, *J* = 12.4 Hz, 1H), 4.04–3.87 (m, 2H), 3.77–3.41 (m, 5H), 3.41–3.28 (m, 2H), 3.00–2.76 (m, 3H), 2.58–2.51 (m, 2H), 2.37–2.29 (m, 1H), 1.90–1.56 (m, 8H); ^13^C NMR (126 MHz) *δ* 163.6, 139.4, 133.7, 133.6, 126.2, 123.4, 115.9, 115.7, 59.8, 59.6, 58.8, 56.7, 54.2 (2), 47.3, 35.3, 31.8, 30.2, 24.1, 24.0, 18.0, 17.9. HRMS: calcd for C_22_H_32_FN_2_O·2HCl [M−2HCl+H]^+^: 359.2493, found: 359.2492.

### Synthesis of (*Z*)-12*N*-(3-nitrobenzyl)-Δ^βγ^-matrinic crotonol dihydrochloride **20**

The compound **12** (1.0 g, 4.0 mmol) was dissolved in 2 N HCl/MeOH (30 mL). The reaction mixture was refluxed for 2 h, then anhydrous K_2_CO_3_ (3.5 equiv) and Boc_2_O (1.5 equiv) were added to the reaction solution, and the mixture solution was stirred at room temperature until TLC analysis showed completion of the reaction. The reaction mixture was filtered, and the filtrate was washed by water and brine, dried with anhydrous Na_2_SO_4_, filtrated and concentrated to afford the crude **15**.

A solution of the LiAlH_4_ in THF (2.4 N, 1.2 equiv) was added to the solution of compound **15** in anhydrous THF in ice-bath, then the mixture solution was stirred at room temperature for 30 min, the reaction was then quenched with acetone, 2 mL saturated ammonium chloride solution was added and stirred for 30 min, and the precipitation was filtrated off. The filtrate was concentrated, and the residue of compound **16** was dissolved in ethyl acetate, and washed with water and brine, dried with anhydrous Na_2_SO_4_, filtrated and concentrated. The residue was stirred in 2 N HCl/Et_2_O (20 mL) to remove the Boc protection group, then the mixute was filtrated to give the crude **17**.

The crude **17** (1.0 equiv), TBSCl (1.2 equiv) and imidazole (1.5 equiv) were used to synthesize compound **18** in CH_2_Cl_2_, after reaction was complete, 3-nitrobenzyl bromide (3.0 equiv) and TEA (3.0 equiv) were added to the reaction solution, which was stirred at room temperature until TLC analysis showed completion of the reaction. The reaction solution was washed by water and brine, dried over anhydrous Na_2_SO_4_, filtrated and concentrated to afford the crude compound **19**.

The crude **19** was dissolved in 2 N HCl (15 mL), and the mixture was stirred until TLC analysis showed completion of the reaction. The pH of the reaction solution was then adjusted to 7–8 by addition of ammonium hydroxide. The solvent was removed under reduced pressure, and the residue was dissolved in MeOH and filtered to remove the organic salts. The solution was concentrated, and the residue was purified by flash column chromatography on silica gel with dichloromethane and methanol as the eluent to afford compound **20** as white solid. Yield: 30%; mp: 143–145 °C; ^1^H NMR (400 MHz) *δ* 12.8 (br, 1H), 11.17 (br, 1H), 8.44 (s, 1H), 8.30 (d, *J* = 8.0 Hz, 1H), 8.06 (d, *J* = 8.0 Hz, 1H), 7.75 (t, *J* = 8.0 Hz, 1H), 6.20–6.14 (m, 1H), 5.73 (t, *J* = 10.4 Hz, 1H), 5.27–5.20 (m,1H), 4.85(d, *J* = 12.8 Hz, 1H), 4.10–4.02 (m, 3H), 3.65 (d, *J* = 10.4 Hz, 1H), 3.57–3.49 (m, 2H), 3.29 (d, *J* = 12.0 Hz, 2H), 3.00–2.88 (m, 3H), 2.60–2.53 (m, 3H), 2.37–2.32 (m, 1H), 1.91–1.56 (m, 8H); ^13^C NMR (126 MHz) *δ* 147.8, 139.7, 138.0, 131.9, 130.4, 126.3, 124.3, 123.3, 59.8, 59.6, 58.9, 56.5, 54.2, 47.6, 35.3, 31.8, 30.3, 29.2, 24.1, 23.9, 18.0, 17.9. HRMS: calcd for C_22_H_32_N_3_O_3_·2HCl [M−2HCl+H]^+^: 386.2438, found: 386.2440.

### Biology assay

#### Cell culture

Human liver cell line Huh7.5 cells (kindly provided by Vertex Pharmaceuticals, Inc., Boston, MA) were cultured in Dulbecco’s modified eagle medium (DMEM) supplemented with 10% inactivated fetal bovine serum and 1% penicillin–streptomycin (invitrogen). Cells were digested with 0.05% trypsin-ethylene diamine tetraacetic acid (EDTA) and split twice a week.

#### Anti-HCV effect in vitro

Huh7.5 cells were seeded into 96-well or 6-well plates (Costar) at a density of 3 × 10^4^ cells cm^−2^. After 24 h incubation, the cells were infected with HCV viral stock (45 IU cell^−1^) and simultaneously treated with the test compounds at various concentrations or solvent as control. The culture medium was removed after 72 h inoculation, the intracellular total RNA (in 96-well plates) was extracted with RNeasy Mini Kit (Qiagen), and total intracellular proteins (in 6-well plates) were extracted with Cyto-Buster Protein Extraction Reagent added with 1 mM protease inhibitor cocktail. The intracellular HCV RNA was quantified with a real time one-step reverse-transcription polymerase chain reaction (RT-PCR).

#### Cytotoxicity assay

Huh7.5 cells were seeded into 96-well plates (Costar) at a density of 3 × 10^4^ cells cm^−2^. After 24 h incubation, fresh culture medium containing test compounds at various concentrations were added. 72 h later, cytotoxicity was evaluated with 3-(4,5-dimethylthiazol-2-yl)-2,5-diphenyltetrazolium bromide (MTT).

### PK studies

Three male SD mice were used in each study. Each of them was dosed with a tested compound at 25 mg kg^−1^ via oral administration. Eight blood samples were respectively collected at 0, 0.25, 0.5, 1.0, 2.0, 4.0, and 6.0 h and were immediately centrifuged to separate the plasma fractions. The separated plasma samples were stored at −20 °C for analysis. Concentration-versus-time profiles were obtained for each analyte, and standard non-compartmental analysis was performed on the data using WinNonlin software, version 5.3, to recover the AUC and other non-compartmental parameters.

### Acute toxicity

Female Kunming mice with weight of 20.0 ± 1.0 g were fed with regular rodent chow and housed in an air conditioned room. The mice were randomly divided into different groups with six mice each. Each compound was given orally in a single-dosing experiment at 0, 250, 500, 750 or 1000 mg kg^−1^ (ddH_2_O as control), respectively. The mice were closely monitored for 7 days. Body weight as well as survival was monitored.

## Conclusion

In conclusion, 32 compounds (of which 27 were novel) with diverse structures, including methyl matrinate, matrinols, matrinic butane, 1′, 1′-dialkylmatrinols, (*Z*)-methyl Δ^βγ^-matrinic crotonates, (*Z*)-Δ^βγ^-matrinic crotonols were synthesized and evaluated for their anti-HCV activities, taking compound **1** as the lead. The SAR study indicated that the introduction of electron-donating substitutions on the benzene ring was helpful for the anti-HCV activity, and the unsaturated 11-side chain might not be favorable for the activity. Out of the gathered compounds, matrinol **7a** demonstrated a potential anti-HCV effect with the SI value of 136. Further study showed that compound **7a** possessed reasonable PK and safety profiles in vivo, indicating a fair druggability nature. Besides, the free hydroxyl arm in **7a** would make it possible to be a parent structure to make pro-drug candidates for their potential in the treatment of HCV infection. This study provided powerful information on further strategic optimization and development of this kind of compounds into a novel family of anti-HCV agents.
